# Impact of different control policies for COVID-19 outbreak on the air transportation industry: A comparison between China, the U.S. and Singapore

**DOI:** 10.1371/journal.pone.0248361

**Published:** 2021-03-16

**Authors:** Fanyu Meng, Wenwu Gong, Jun Liang, Xian Li, Yiping Zeng, Lili Yang

**Affiliations:** 1 Department of Statistics and Data Science, Southern University of Science and Technology, Shenzhen, People’s Republic of China; 2 Academy for Advanced Interdisciplinary Studies, Southern University of Science and Technology, Shenzhen, People’s Republic of China; 3 School of International Development, University of East Anglia, Norwich, United Kingdom; University of Rochester, UNITED STATES

## Abstract

Many countries have been implementing various control measures with different strictness levels to prevent the coronavirus disease 2019 (COVID-19) from spreading. With the great reduction in human mobility and daily activities, considerable impacts have been imposed on the global air transportation industry. This study applies a hybrid SARIMA-based intervention model to measure the differences in the impacts of different control measures implemented in China, the U.S. and Singapore on air passenger and air freight traffic. To explore the effect of time span for the measures to be in force, two scenarios are invented, namely a long-term intervention and a short-term intervention, and predictions are made till the end of 2020 for all three countries under both scenarios. As a result, predictive patterns of the selected metrics for the three countries are rather different. China is predicted to have the mildest economic impact on the air transportation industry in this year in terms of air passenger revenue and air cargo traffic, provided that the control measures were prompt and effective. The U.S. would suffer from a far-reaching impact on the industry if the same control measures are maintained. More uncertainties are found for Singapore, as it is strongly associated with international travel demands. Suggestions are made for the three countries and the rest of the world on how to seek a balance between the strictness of control measures and the potential long-term industrial losses.

## Introduction

The spread of the severe acute respiratory syndrome coronavirus 2 (SARS-Cov-2) has caused an extensive outbreak of a global pandemic of COVID-19 [1]. The disease first emerged in late Dec, 2019 in mainland China, and rapidly spread through Asia, Europe, North America to the rest of the world, upon which the World Health Organization (WHO) has declared it to be a pandemic on Mar 11 [2]. Up to Apr 3, 2020, the number of global confirmed cases has reached one million, and the WHO has more than once urged relevant countries to implement containment measures and suggested effective measures at different stages of the pandemic situation [3, 4]. As it has come to a global consensus based on the experiences from previous epidemic control that human mobility is the key to the disease transmission, studying mobility patterns during the COVID-19 epidemic has become of utmost importance. Wang et al. [5] found a 14-day delay between people’s awareness of the pandemic and change in mobility patterns of the U.S. Kang et al. [6] proposed that the most effective factor that affects population mobility is the governmental non-pharmaceutical intervention strategies. Lee et al. [7] has modeled spatial-temporal heterogeneity of human mobility in early stages of the pandemic in the U.S. and confirmed the effectiveness of the staying home order. Similarly, Galeazzi et al. [8] also identified a massive reduction in mobilities in France, Italy and the U.K., respectively after lockdown measures. In fact, control measures such as travel restrictions, city lock-down, cordon sanitaire, and night curfew have been enforced in many countries. Besides, quarantine policies, closures of schools and stores, and social distancing were also implemented in many places of the world to minimize the spreading rate of the virus [9, 10].

Such control measures are effective in confining the disease, yet they also place severe impacts on travel-related industries at the same time. The air transportation industry, profiting mainly from providing transportation services for passengers and goods, have been impacted directly by the confinements on human traveling and indirectly by the restricted human activities. According to the predictions by International Air Transport Association (IATA), the COVID-19 pandemic may cause a total loss of 21.5 billion USD in 2020 for European airlines, and the predicted losses for Asia Pacific airline markets range from 47 billion to 57 billion USD for different scenarios of COVID-19 evolvements [11, 12]. On the supply side, international flights were drastically cancelled following the travel restrictions from regions with high risks starting from Jan, followed by cancellation of domestic flights in China, European countries and the U.S. to prevent the virus from transmission domestically [13]. On one hand, the cancellation of flights has led to direct reduction in air passenger transportation; on the other hand, the reduced belly cargo ability resulted in a recession in international and domestic cargo transfer to a great extent. On the demand side, closures of schools, tourism spots and business areas have cut the travel demand of people, and the deactivated retail markets have lowered the supply chain demand, and thus have weakened the vitality of the entire global air transportation industry.

The effectiveness of control measures on the spread of the disease is influenced by two factors: the strictness of the control policies and the duration for the policies to be in force [1]. Major affected countries by the pandemic have policies with different strictness levels regarding epidemiological control to balance the evolvement of the disease situation with social and economic losses. For the air transportation industry, strict control policies are certainly effective in ‘flattening the curve’ of the number of confirmed cases against time and shortening the post-outbreak recovery period, but might cause a steep decrease in the air passenger and cargo numbers in a short period of time; less strict control measures, on the other hand, may shortly incur fewer losses in the revenue of the industry, but need to be implemented for a longer time to achieve ‘herd immunity’, probably with more accumulative social losses [13]. Besides, the duration that a certain industry to be suppressed for can also affect its recovery behavior. In a word, predicting the industrial losses is subject to quantitatively understanding the trade-offs between the strictness of different types of control policies and the duration that certain policies need to be implemented to be able to constrain local epidemic situation. Therefore, it is vital to quantitatively assess the differences in the impacts of different control policies (or confinement concepts) on the air transportation industry under different effective periods to guide the authorities to find the balance for their own benefits.

This study aims to predictively evaluate the impacts of different control measures for COVID-19 on the air transportation industry in a comparative manner. With that said, time series intervention analyses are carried out with a comparison between China, the U.S. and Singapore, provided that the three countries have distinctively implemented different control policies (with different strictness levels) at different times dealing with COVID-19 (see S1 File as the timelines of the major control policies assumed to affect the air transport industry). To study the sensitivity of the time span of control policies enforcement (i.e., the effect of the duration of the confinement), two intervention scenarios are defined: (1) short-term intervention (policies effective for 3 or 6 months) and (2) long-term intervention (policies effective till the end of 2020). Three forms of interventional transfer function terms are defined and compared as different forms of externalities to the time series function and the optimal one is chosen for the final predictions. Predicted losses in the industry for the scenarios of the three countries are calculated and discussed accordingly. According to the model results, China is predicted to undergo a relatively milder impact in air transportation industry in the long run, while Singapore and the U.S. would suffer a deeper and more confounding effects from the confinements. Suggestions are made in the Discussions for dealing with similar situations in the future based on the predicted results of the hypothesized intervention scenarios, with the rationale to keep a balance between industrial losses and domestic epidemic situations.

## Data and methods

### Data description

Monthly air passenger and air freight traffic/turnover data for China, Singapore and the U.S. between Jan 2010 and May 2020 were collected from Census and Economic Information Center (CEIC) Dataset (https://insights.ceicdata.com/). The CEIC Dataset has collected economic data from official data releasements from relevant autonomies in the world, where the collected indicators can represent the travel demands a country’s aviation industry [14]. In this paper, air passenger traffic (in million) and air freight traffic (in million tons) were used for the predictions for all three countries, except that the air freight turnover (in million ton-miles) was used as the U.S. air freight indicator, as the Federal Aviation Administration did not release latest air freight traffic information in weights.

### Model selection

Time series forecasting is the model applied to predict future values based on previously observed values and related methods can be classified into models with linear dependence and those with non-linear dependence [15]. There are three basic model classes with linear dependence, namely autoregressive (AR) models, integrated (I) models and moving average (MA) models, the combination of which, ARIMA, is the most commonly used model to explain the constant variations of a time series [16]. Besides, a set of extensions of ARIMA models, such as seasonal ARIMA (SARIMA) [17] and ARIMA exogenous (ARIMAX) models [18], are also applied to represent some specific features of time series analyses, in which the former mainly addresses the seasonality feature in the time series and the latter focuses on the exogenous factors. The non-linear dependence of a series on previous data points is also of interest, partly because of the possibility to produce a chaotic time series and the change of variance over time [19]. The most frequently used non-linear time series model is the autoregressive conditional heteroskedasticity (ARCH) model family [20–23]. The ARCH models are efficient to deal with heteroskedasticity in time series and commonly employed to modeling financial time series that exhibits time-varying volatility and volatility clustering [24–26], i.e. periods of swings interspersed with periods of relative calm [27].

The characters of time series are also possible to be disturbed by external events, often referred to as interventions, and hence intervention analysis is introduced to solve the cases where the classical time series approaches fail. In time series analyses with interventions, the most important task is the estimation of intervention which is referred to as transfer function, mainly based on ARIMA models [28]. Such intervention analysis has been proven capable of forecasting the tourism demand or airport arrivals under the influence of epidemic outbreaks. Min [29] and Joo [30] have applied intervention models to assess the impacts of epidemic (SARS and MERS) on inbound tourism. Besides, forecasting airport passenger arrivals have also been made based on SARIMA intervention models [31].

Similar to tourism demand and airport passenger arrivals, air passenger and cargo demands are assumed to have a seasonal feature with linear dependence [32–35]. To evaluate the impacts of different control policies on the air transportation industry, methods that can forecast air passenger and air freight traffic/turnover based on historical data are desired to be implemented. In addition, the multiple scenarios with different intervention periods are also designed. Hence, in this paper, upon testing the seasonality and linearity of dependence of the data, a SARIMA-based intervention model, the systematic combination of SARIMA fluctuation model and intervention model with exogenous variables, is used to identify the impacts of control measures on the air transportation industry in the three selected countries. Linear/non-linear intervention forms are compared to better delineate sensitivity of the studied index to the control policies enforcement. The rest of this section introduces the data used for modeling and the model framework of the SARIMA intervention analysis.

### SARIMA fluctuation model

The stationary process, meaning that the mean and covariance of the series are not change over time, is essential in time series modeling. There are three basic stationary time series models: auto-regressive (AR), moving average (MA), and auto-regressive moving average (ARMA) models. We assume that F_t_ denotes the forecasting values at time *t*; *X*_*t*_ is the observed values at time *t*; and *ϵ*_*t*_ represents the current residual. The basic time series ARMA(*p*,*q*) model have the following mathematical form [31]:

$$\begin{array}{c} F_{t}=\phi_{1}X_{t-1}+\phi_{2}X_{t-2}+\cdots +\phi_{p}X_{t-p}+\epsilon_{t}\\ +\epsilon_{t}-\theta_{1}\epsilon_{t-1}+\theta_{2}\epsilon_{t-2}+\cdots +\theta_{q}\epsilon_{t-q} \end{array}$$ (1)

where *ϕ*_*p*_ is the *p*th auto-regressive parameter and *θ*_*q*_ is the *q*th moving average parameter. Transformation of the series into a stationary one, such as differencing, has to be performed first for non-stationary time series. Then, the differenced stationary time series can be modeled as ARMA(*p*,*q*) model to yield ARIMA(*p*,*d*,*q*) model. The new notation *d* denotes the orders of difference required by the original process.

Seasonal fluctuation in time series has been found to effectively account for the time series non-stationarity [36], which can be modeled by the seasonal ARIMA (i.e. SARIMA) model. Dickey-Fuller tests are performed for all datasets to identify seasonal unit roots, and the insignificant results indicate that the time series are non-stationary (see S1 Table). Hence, seasonal differencing is necessary to convert the time series to be seasonally stationary. After the seasonal decomposition, the series of monthly air passenger and air freight traffic/turnover data became stationary (see S2 File), so the SARIMA model (Eq 2) is applied to deal with the non-stationary time series.

$$\begin{array}{c} {\left(1-B\right)}^{d}(1-B^{s}{)}^{D}F_{t}=\frac{\theta \left(B\right)\Theta (B^{s})}{\phi \left(B\right)\Phi (B^{s})}\epsilon_{t},\\ \begin{array}{cc} \phi (B) & =1-\phi_{1}B-\phi_{2}B^{2}-\cdots -\phi_{p}B^{p}\\ \Phi (B^{s}) & =1-\Phi_{1}B^{s}-\Phi_{2}B^{s2}-\cdots -\Phi_{P}B^{sP}\\ \theta (B) & =1-\theta_{1}B-\theta_{2}B^{2}-\cdots -\theta_{q}B^{q}\\ \Theta (B^{s}) & =1-\Theta_{1}B^{s}-\Theta_{2}B^{s2}-\cdots -\Theta_{Q}B^{sQ} \end{array} \end{array}$$ (2)

where *ϕ*(*B*) is the nonseasonal AR operator, *θ*(*B*) the nonseasonal MA operator, Φ(*B*^*s*^) the seasonal AR operator, Θ(*B*^*s*^) the seasonal MA operator, *B* the back-shift operator. (1 − *B*)^*d*^ is the nonseasonal *d*th differencing, and (1 − *B*^*s*^)^*D*^ the seasonal *D*th differencing at *s* number of lags, where the order of seasonality *s* equals 12 months. *p* is the order of nonseasonal AR process; *P* is the order of seasonal AR process; *q* is the order of nonseasonal MA process; and *Q* is the order of seasonal MA process. These notations can be represented by SARIMA(*p*, *d*, *q*)(*P*, *D*, *Q*)_*s*_, where the parentheses enclose the nonseasonal and seasonal parameter, respectively.

Generally, heteroskedasticity tests are suggested to be performed on the residuals of the time series regression to ensure there isn’t nonlinear dependence in the series, among which White test is one of the most classic methods [37]. Results of the White Test for the air passengers and freight series in the three countries (see S2 Table) indicate that there is no significant heteroskedasticity in the residuals of the SARIMA model in this study. This paper used the Python version 3.6 and Box-Jenkins modeling steps [38] to perform the SARIMA models’ identification, estimation, diagnostic checking and forecasting.

### SARIMA intervention analysis

Intervention analysis, which aims at measuring the impact caused by external shock or exogenous intervention occurred at some identifiable time points, was originally developed by Box and Tiao [39]. Based on a SARIMA modeling framework, the intervention impact assessment model can be written as:

$$\begin{array}{c} f\left(F_{t}\right)=g\left(k,\xi ,t\right)+N_{t}, \end{array}$$ (3)

where: *f*(F_t_) is an appropriate transformation of F_t_, such as *log*(F_t_) or *exp*(F_t_); *g*(*k*, *ξ*, *t*) can allow for intervention effects represented by *k* exogenous variables *ξ* at time *t*; *N*_*t*_ represents stochastic background variation, which is a SARIMA structure in this paper.

In intervention analysis, the types of impact from relevant intervention should be very carefully addressed by all exogenous indicator variables. In general, the exogenous indicator variables *ξ*_*t*_ take the values 0 and 1 to denote the non-occurrence and occurrence of certain interventions. There are two general types of indicator variables used to represent the two kinds of basic interventions: a step intervention (${S_{t}}^{T}$) and a pulse intervention (${P_{t}}^{T}$) as shown in Eq 4, where the intervention appears at time *T* [29].

$$\begin{array}{c} {S_{t}}^{T}=\left\{ \begin{array}{c} \begin{array}{ccc} 0 & & t<T\left(\text{before}\;\text{the}\;\text{intervention}\right)\\ 1 & & t\geq T\left(\text{at}\;\text{and}\;\text{after}\;\text{the}\;\text{intervention}\right) \end{array} \end{array},\right. \end{array}$$ (4)

$$\begin{array}{c} {P_{t}}^{T}=\left\{ \begin{array}{c} \begin{array}{ccc} 0 & & t\neq T\left(\text{not}\;\text{at}\;\text{intervention}\right)\\ 1 & & t=T\left(\text{at}\;\text{intervention}\right) \end{array} \end{array}.\right. \end{array}$$ (5)

${S_{t}}^{T}$ describes a lasting effect and ${P_{t}}^{T}$ represents a relatively temporary change that only affects the very moment after the intervention appears. Besides, the step and pulse interventions are interrelated, and can be expressed as:

$$\begin{array}{c} {P_{t}}^{T}=\left(1-B\right){S_{t}}^{T}. \end{array}$$ (6)

Based on the two scenarios with different time spans of the control policies, different intervention functions have been explored. The explanatory form of intervention effects, based on the two kinds of basic intervention variables, can commonly be expressed as either a linear or a non-linear function [29]. Note that the SARIMA intervention model with a linear exogenous intervention term is the same with the form of seasonal ARIMAX (SARIMAX) model [18]. For easy representation, we suppose that there is one single intervention variable in this case (*k* = 1).

- Linear intervention functions (SARIMAX).
  (1) Short-term intervention:
  $$g_{1}\left(1,\xi ,t\right)=\sum_{T=1}^{m}{P_{t}}^{T},m=3\;or\;6,$$ (7)
  (2) Long-term intervention:
  $$g_{1}\left(1,\xi ,t\right)={S_{t}}^{T}.$$ (8)
- Non-linear intervention functions. According to Box and Tiao [39], there are many non-linear intervention functions which can interpret the intervention effects with the two basic intervention variables. The choice of intervention functions is determined visually by SARIMA residual graphs during the period of intervention. In this paper, we accordingly defined the short-term intervention scenario in three-parameter and two-parameter non-linear intervention function forms, which can be addressed by Eqs 9 and 10 respectively. And the long-term intervention scenario is formulated by Eq 11.
  (1) Short-term intervention:
  $$g_{2}\left(1,\xi ,t\right)=\frac{\omega_{0}-\omega_{1}B}{1-\delta B}\left({S_{t}}^{T}-{S_{t}}^{T+m}\right),m=3\;or\;6,$$ (9)
  or
  $$g_{2}\left(1,\xi ,t\right)=\sum_{T=1}^{m}\frac{\omega_{1}B}{1-\delta B}{P_{t}}^{T},m=3\;or\;6,$$ (10)
  (2) Long-term intervention:
  $$g_{2}\left(1,\xi ,t\right)=\frac{\omega_{1}B}{1-\delta B}{S_{t}}^{T}.$$ (11)

The SARIMA intervention model can be treated as an extension of SARIMA model, which serves as a useful dynamic modeling tool to rigorously analyze the impact of interventions [40]. And a series of literatures have shown that there is an improvement in the quality of forecasts when intervention analysis is used to capture the impact of a significant intervention [31, 41]. The general form can be expressed by Eq 12.

$$\left(1-B\right)(1-B^{12})F_{t}=\left(1-B\right)(1-B^{12})g_{l}\left(1,\xi ,t\right)+\frac{\theta \left(B\right)\Theta (B^{12})}{\phi \left(B\right)\Phi (B^{12})}\epsilon_{t},\;l=1,2.$$ (12)

During the COVID-19 outbreak, the time of negative impact of the control measures on the selected industrial indicators started to take effect varies in different countries. Thus, the proposed SARIMA intervention model was used to measure the effect of short-term and long-term interventions. Given the nature of intervention model, the SARIMA parameters were initially computed prior to the modeling of intervention model and the intervention effects were programmed by SAS version 9.4 and guided by Box-Jenkins modeling steps [38].

### Comparisons of forecasting performances between intervention model forms

In this paper, linear and non-linear intervention models have been introduced. To choose the model forms, the observed values of the original series is compared with the projected values obtained through the linear and non-linear intervention models, respectively. To evaluate the performances of the SARIMA intervention models, there are a number of ways to measure the models’ forecasting abilities, and the most commonly used are mean absolute percentage error (MAPE), mean absolute error (MAE) and root mean square error (RMSE) [34]. Just as shown in Eqs 13, 14 and 15, where *F*_*t*_ is the projected value at time t; *X*_*t*_ is the observed value at time t; and *T* is the calculation period specified for the calculation of the metric.

$$\begin{array}{c} \begin{array}{c} \text{MAPE}=\frac{1}{T}\sum_{t=1}^{T}\left|\frac{F_{t}-X_{t}}{X_{t}}\right|, \end{array} \end{array}$$ (13)

$$\begin{array}{c} \begin{array}{c} \text{MAE}=\frac{1}{T}\sum_{t=1}^{T}\left|F_{t}-X_{t}\right|, \end{array} \end{array}$$ (14)

$$\begin{array}{c} \begin{array}{c} \text{RMSE}=\sqrt{\frac{1}{T}\sum_{t=1}^{T}{\left(F_{t}-X_{t}\right)}^{2}}. \end{array} \end{array}$$ (15)

Based on the optimal SARIMA model structures (see SARIMA estimations in S3 Table), the forecasting performances of linear and non-linear intervention models are compared in S5 Table (taking 6-month intervention as an example). The three above-mentioned performance indicators were calculated based on only the intervention period. All the values of indicators (namely MAPE, MAE and RMSE) in the nonlinear intervention model were lower than those of the corresponding linear intervention model. These results show that the non-linear intervention models generally have a better performance in forecasting air passengers and air freight traffic for all the selected countries.

For short-term intervention, the performances of the three-parameter and the two-parameter forms are also compared using MAPE, MAE and RMSE. The error indicators values in S4 and S5 Tables show that the performances of three-parameter intervention models have a better performance than their two-parameter counterparts. (Note that the three-parameter model form for 6-month intervention was not suitable for Singapore air passengers and China air freight, as convergence of these models was not reached). Based on these model comparisons, the following Tables 1 and 2 presents our final choice of intervention models for 3-month and 6-month short-term interventions, respectively.

**Table 1:** Intervention model choices for 3-month short-term intervention.

Indicators	Linear intervention	Non-linear intervention	
		Three-parameter	Two-parameter
China air passengers	×	×	√
U.S. air passengers	×	√	×
Singapore air passengers	×	√	×
China air freight	×	×	√
U.S. air freight	×	√	×
Singapore air freight	×	×	√

√ denotes the selected model form.

**Table 2:** Intervention model choices for 6-month short-term intervention.

Indicators	Linear intervention	Non-linear intervention	
		Three-parameter	Two-parameter
China air passengers	×	√	×
U.S. air passengers	×	√	×
Singapore air passengers	×	×	√
China air freight	×	×	√
U.S. air freight	×	√	×
Singapore air freight	×	√	×

√ denotes the selected model form.

## Results

### Estimation results for SARIMA intervention models

Based on the SARIMA intervention model choices shown in Tables 1 and 2, the estimation results of the two intervention scenarios are shown in Tables 3 and 4, respectively. The residual plots of the fitted model (shown in S3 File) indicate that all of the models are adequate to predict the selected indicators with intervention.

**Table 3:** SARIMA intervention results for 3-month short-term intervention.

	Estimated coefficients						Model performance		
Indicators	AR(1)	MA(1)	SMA(12)	*ω*_0_	*ω*_1_	*δ*	Adj-*R*^2^	AIC	MAPE (%)
China air passengers	-	0.9077	0.2428	-	-19.475	0.829	0.95	503.8	3.84
U.S. air passengers	-	0.7241	-0.5158	-38.531	-22.980	0.752	0.99	323.1	1.32
Singapore air passengers	0.3750	0.7883	-	-0.462	0.474	0.769	0.98	-395.7	3.11
China air freight	-0.3797	0.7748	-	-	-0.0683	0.801	0.89	-457.6	4.27
U.S. air freight	-0.3481	-	0.6298	-203.699	239.825	-0.647	0.96	929.7	2.41
Singapore air freight	-	0.2155	0.6179	-	0.0117	-0.456	0.61	-793.8	4.27

*D* and *d* are both equal to 1, and all estimated coefficients are significant at the 0.05 significance level.

**Table 4:** SARIMA intervention results for 6-month short-term intervention.

	Estimated coefficients						Model performance		
Indicators	AR(1)	MA(1)	SMA(12)	*ω*_0_	*ω*_1_	*δ*	Adj-*R*^2^	AIC	MAPE (%)
China air passengers	-	0.7775	0.4601	-5.567	42.877	-0.211	0.98	385.9	2.74
U.S. air passengers	-	0.7241	-0.5158	-38.531	-22.980	0.752	0.99	323.1	1.32
Singapore air passengers	-0.8034	-0.8807	-	-	-0.649	0.812	0.94	-282.5	4.56
China air freight	-0.3797	0.7748	-	-	-0.0683	0.801	0.88	-457.6	4.41
U.S. air freight	-0.3481	-	0.6298	-203.699	239.825	-0.647	0.96	929.7	2.41
Singapore air freight	-	0.6657	0.5828	0.0125	0.0476	0.487	0.88	-905.3	3.25

*D* and *d* are both equal to 1, and all estimated coefficients are significant at the 0.05 significance level.

As shown in Tables 3 to 5, all twelve models have the overall acceptable model performances with all adjusted-*R*^2^ higher than 0.6, and the overall predictability with the acceptable MAPE values [18, 41]. From the estimated results, it can be concluded that the studied indicator for all three countries under both two scenarios have a strong seasonality with a 12-month cycle. The estimated coefficients for the AR(1), MA(1) and SMA(12) terms are significant at the 0.05 level and smaller than 1 in all estimated models, which echoes with the required stationarity and invertibility assumptions of SARIMA modeling [42]. Besides, the estimated coefficients for the intervention terms are significant at the 0.05 level (i.e. *ω*_0_, *ω*_1_ and *δ*), indicating that all indicators are significantly affected by the corresponding control measures issued in the three countries.

**Table 5:** SARIMA intervention results for long-term intervention.

	Estimated coefficients					Model performance		
Indicators	AR(1)	MA(1)	SMA(12)	*ω*_1_	*δ*	Adj-*R*^2^	AIC	MAPE (%)
China air passengers	-	0.3018	0.5804	-44.143	-0.147	0.98	400.8	2.82
U.S. air passengers	-	0.9629	-0.4056	-43.409	0.453	0.98	369.6	3.23
Singapore air passengers	-0.8034	-0.8806	-	-0.649	0.8119	0.94	-282.5	4.56
China air freight	-0.3688	0.7810	-	-0.0912	0.356	0.88	-453.9	4.42
U.S. air freight	-0.5474	-	0.5627	-235.771	0.196	0.95	949.9	2.11
Singapore air freight	-	0.0706	0.6313	0.0213	-0.417	0.87	-796.3	3.43

*D* and *d* are both equal to 1, and all estimated coefficients are significant at the 0.05 significance level.

**Fig 1:**
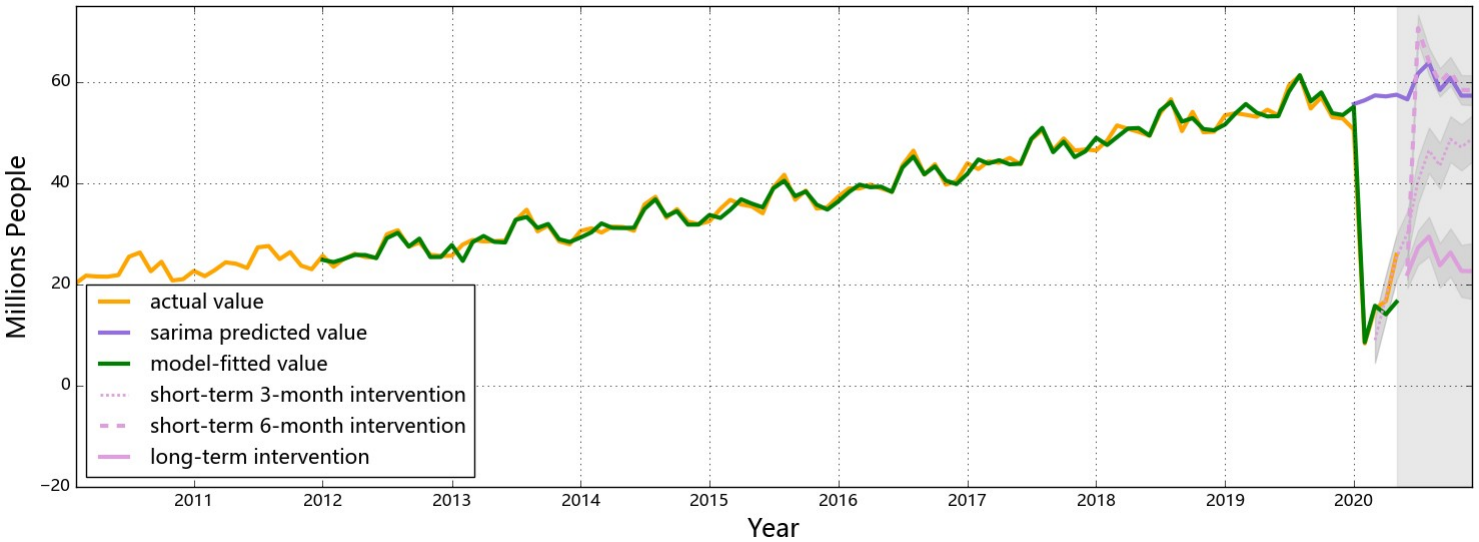
Predictions for air passengers in China. The observed data dropped from 50.6 million in Jan, 2020 to 8.3 million in Feb of the same year. For the short-term intervention scenario (as shown by the solid pink line), air passenger will rebound to 74.1 million in Aug and then gradually drop back to the level as the SARIMA predictions (i.e. the purple line).

**Fig 2:**
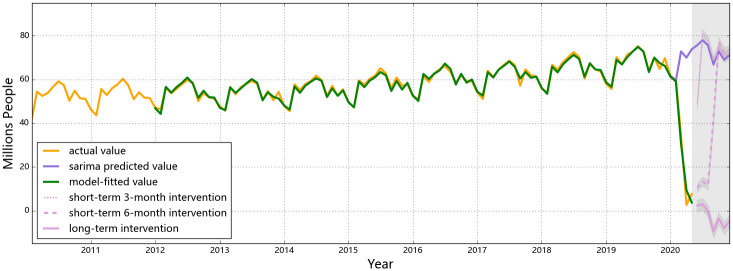
Predictions for air passengers in the U.S. An obvious decline in the number of American air passenger is observed from Feb to Apr, 2020 (from 59.8 to 2.6 million), and then the number fluates to 7.6 million in May. The number will keep dropping to 0 level if the intervention is valid till the end of 2020 (shown as the pink dashes), yet a rebound will occur in Sep at the end if the intervention ends after six-month (shown as the solid pink line).

**Fig 3:**
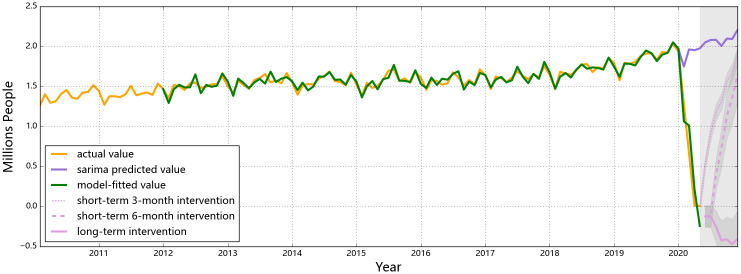
Predictions for air passengers in Singapore. The drop of the observed number of air passengers in Singapore is almost linear at the beginning of 2020, and the value has reached 0.009 million in Apr. The hybrid model predicts that the decreasing trend of air passenger traffic will last till the end of 2020, but the actual numbers will be at thousand level. The air passenger is predicted to be unable to recover to the normal level (the purple line) within this year for both short-term and long-term interventions.

**Fig 4:**
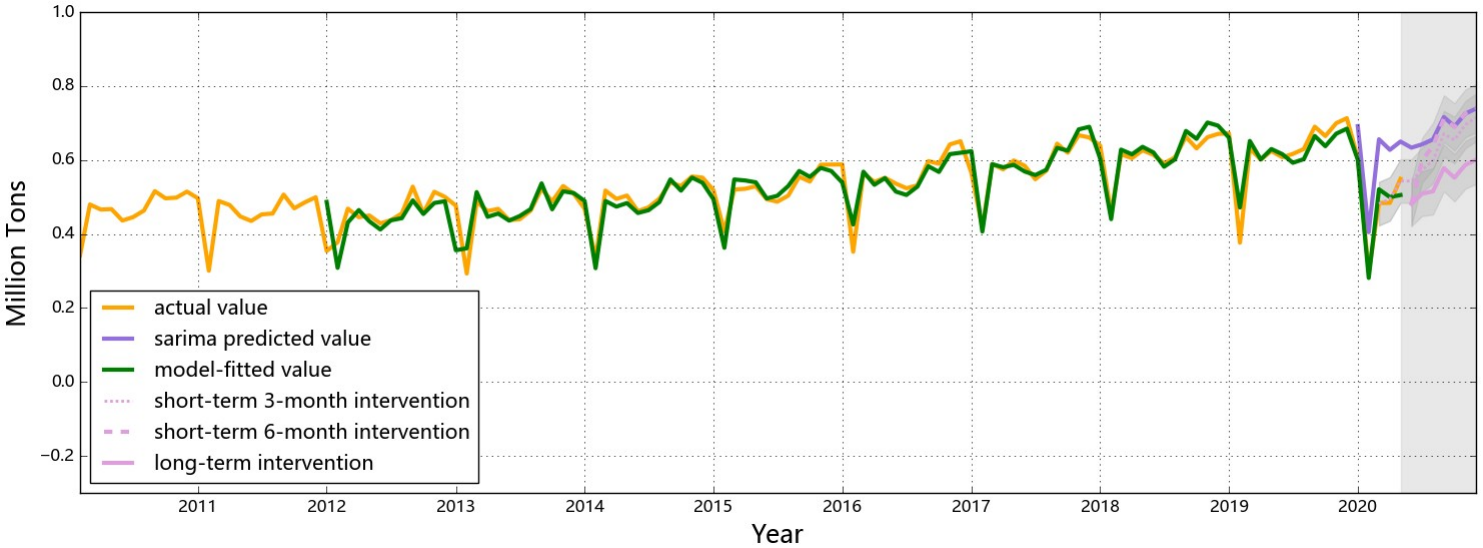
Predictions for air freight in China. China air freight was mostly impacted in Feb, Mar and Apr, 2020, but the distances between the predicted values without intervention (the purple line) and the actual values (the yellow line) are almost the same for these three months (i.e. 0.12, 0.14 and 0.13 for the three months, respectively). The numbers are predicted to rebound to normal level once the intervention ends in Jun (as shown by the solid pink line).

**Fig 5:**
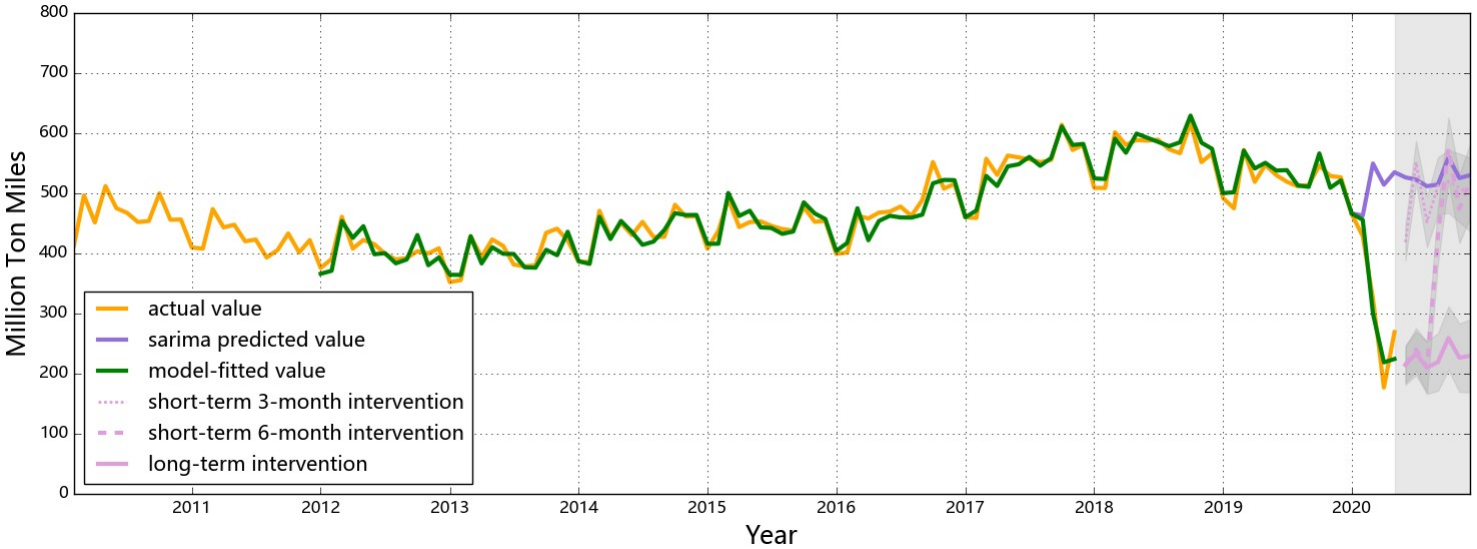
Predictions for air freight in the U.S. The lowest point of air freight turnover in the U.S. was in Apr, 2020 at 177.0 million ton-miles. For the short-term intervention, the number will quickly rebound to 524 million ton-miles in Sep (shown as the solid pink line). If the same control policies last the whole year, the numbers will fluctuate around 230 million ton-miles till yearend.

**Fig 6:**
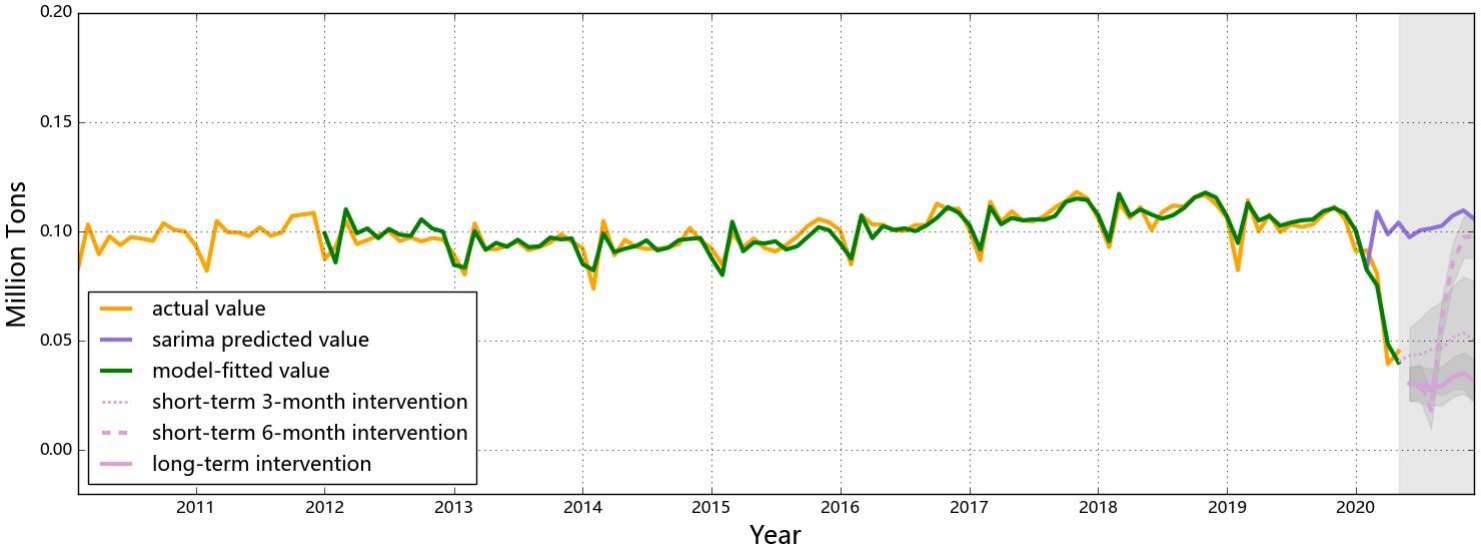
Predictions for air freight in Singapore. The actual air freight traffic in Singapore has been dropping from Feb (0.081 million tons) till Apr, 2020 (0.039 million tons). Since it stops decreasing from May, the number will keep fluctuating if the same policies last till the end of the year. Nonetheless, air freight will not recover to the normal level (represented by the purple line) for both scenarios.

The observed and the predicted values of air passenger and air freight indicators for all three countries are shown in Figs 1 to 6, respectively. The predicted values form the two hypothesized intervention scenarios have been plotted in the same figure with different types of lines to display the different trends of the indicators under different time spans of intervention. To reflect the levels of effects of the intervention scenarios based on the forecasting ability of the SARIMA intervention models, the percentage changes of all six entities from the start of the intervention compared to the same time of 2019 are presented in Tables 6–8.

**Table 6:** Relative changes (%) compared to 2019 for 3-month intervention.

Indicators	Jan.	Feb.	Mar.	Apr.	May.	Jun.	Jul.	Aug.	Sept.	Oct.	Nov.	Dec.	total
China air passenger	-9	-85	-74	-69	-56	-47	-34	-27	-26	-20	-18	-15	-40
U.S. air passenger	-	-	-53	-96	-90	35	4	3	2	2	1	1	-26
Singapore air passenger	-	-27	-66	-100	-100	-74	-56	-43	-34	-25	-20	-14	-50
China air cargo	-12	-27	-26	-21	-16	-14	-11	-9	-6	-5	-4	-3	-12
U.S. air cargo	-	-	-39	-64	-47	-17	11	-7	5	-3	2	-1	-16
Singapore air cargo	-	-9	-2	-64	-59	-59	-56	-55	-54	-50	-50	-55	-48

**Table 7:** Relative changes (%) compared to 2019 for 6-month intervention.

Indicators	Jan.	Feb.	Mar.	Apr.	May.	Jun.	Jul.	Aug.	Sept.	Oct.	Nov.	Dec.	total
China air passenger	-9	-85	-74	-71	-55	-64	-59	16	-8	-2	-1	-1	-32
U.S. air passenger	-	-	-53	-96	-90	-86	-83	-85	-38	6	4	3	-53
Singapore air passenger	-	-27	-66	-100	-100	-99	-106	-81	-63	-46	-35	-25	-68
China air cargo	-12	-27	-26	-23	-16	-24	-7	-3	0	0	0	0	-11
U.S. air cargo	-	-	-39	-64	-47	-41	-37	-36	7	27	15	8	-20
Singapore air cargo	-	-9	-2	-64	-55	-71	-70	-72	-38	-17	-9	-9	-38

**Table 8:** Relative changes (%) compared to 2019 for long-term intervention.

Indicators	Jan.	Feb.	Mar.	Apr.	May.	Jun.	Jul.	Aug.	Sept.	Oct.	Nov.	Dec.	total
China air passenger	-9	-85	-74	-71	-55	-61	-56	-54	-59	-57	-60	-60	-58
U.S. air passenger	-	-	-53	-96	-90	-97	-96	-100	-114	-105	-112	-107	-97
Singapore air passenger	-	-27	-66	-100	-100	-99	-106	-112	-121	-120	-123	-118	-101
China air cargo	-12	-27	-26	-23	-16	-24	-21	-21	-19	-20	-19	-19	-20
U.S. air cargo	-	-	-39	-64	-47	-57	-53	-57	-55	-51	-55	-54	-53
Singapore air cargo	-	-9	-2	-64	-55	-71	-70	-72	-71	-68	-67	-71	-58

### Impacts from long-term control measures

For air passengers, different patterns between China, Singapore and the U.S. are found if the intervention last till the end of 2020 (see Figs 1 to 3). China has taken the lead to implement confinements to control the domestic outbreak of the epidemic starting from late Jan to early Feb by which time the whole world had no clue about details of the virus and the disease (Fig 1). Shortly after, the rest of the world commenced to cancel international flights from China and prohibit travelers from Hubei/China from entering. Hence, a drop in Chinese air passengers were observed in Jan followed by a drastic plunge (a decrease by 83.5%) in Feb. Owing to the strict lock-down measures (influencing the movements of over 40 million people [43]) and the domestic and international travel bans, the situation of the epidemic in China was controlled to a safe level by the end of Feb [44]. Air passengers in China began to rebound starting from Apr, as the needs for domestic business travel started to increase and more domestic flights recovered to normal operations conditionally. The number of air passengers in China is predicted to keep fluctuating seasonally at the current level till the end of this year if the control policies keep consistent through Dec. Compared to 2019, the decrease in monthly numbers of air passengers in China would fluctuate between 54% to 61% in the second half of 2020. A total loss of 409.1 million passengers will be reached in 2020 (incurring a 58.4% annual decrease compared to the predictions from the SARIMA model), leading to an approximately 605.7 billion CNY (87.0 billion USD) revenue loss in the industry [45].

The air passenger number of the U.S. began to drop in Feb (Fig 2), under the influence of its restrictions to and from certain high-risk regions in the world [44]. Along with its domestic outbreak of the COVID-19 in Mar and Apr, travel restrictions have been issued from the U.S. by other countries in the world, and both international and domestic flights have been canceled to a great extent [46]. The largest percentage decrease in air passengers for the U.S. appeared in Apr (i.e. a 92% decrease relative to Mar), indicating that the impact from domestic and international restrictions in traveling still remained severe in Apr. If the same control measures are implemented till the end of 2020, a descending trend shall be observed and the summer air travel peak in the U.S. (from Jun to Aug) would not occur. (Although the theoretically predicted numbers of air passengers will drop to a negative value according to the intervention model, it will practically be at the zero level and will not rebound until the intervention is withdrawn). The year-on-year annual loss in air passengers in the U.S. would reach 97% compared to the previous year. An estimated loss on air passenger in the U.S., for this specific scenario of control policies, is 676.9 million, rendering an approximate revenue loss of 121.1 billion USD [47]. Similar patterns to the U.S. air passengers are found for Singapore: a stark drop from Feb to Apr is observed and the values almost hit zero in Apr and May (Fig 3). Provided that all flights passing through Singapore are international flights, the pandemic and corresponding confinements on traveling all over the world would keep affecting its air passenger transport till the end of the year.

For air cargo transportation, China once again had a distinct pattern in terms of the observed impact from the intervention and the predicted trends if the intervention pertains for the rest of the year, compared to the other selected countries (Fig 4). As a special seasonality feature for the activeness of Chinese business markets, a drop in Jan and an annual low point in Feb are observed for the country’s cargo intensity each year. Since the fiscal year ends in Dec, most trades are concentratively completed by the end of the year and the market enters a relatively inactive mood and recovers after Spring Festival [44]. Hence, the domestic need for commodity logistics usually drops in Feb each year, not an exception for 2020. However, a deeper ‘V-shape’ is found in the first season of 2020, indicating that the air freight demand and capacity has been affected by both travel restrictions and the diminishing demand from consumers. In the first and second month since the outbreak of the pandemic, third-party logistics in China has been impacted severely since local travel controls have been widely applied based on different local response levels, especially for those areas with high risks for COVID-19 outbreak [48]. Nevertheless, the rebound in Mar indicates that air freight in China has been under recovery (even though the actual value did not reach either the predicted value of the SARIMA model or the level at the same time of the previous year), owing to the raising demand for transferring medical equipment to cope with the disease. China has the second largest pharmaceutical industry globally with more than 4000 pharmaceutical manufacturers by the end of 2019 [49]. Facing the urgent demand for fighting the unknown epidemic, the Chinese government has taken the lead to actively stimulate its productivity of medical supplies such as masks and protective suits and has deployed them to the areas with the highest demands, which resulted in an increase in its air cargo demands. A slight ascending trend would occur with monthly fluctuations if similar control measures last to the end of the year, probably resulting from (1) the seasonal lift in the number and/or (2) the gradual recovery of local businesses. Compare to 2019, the percentage change in the monthly air cargo tonnages in the second half of 2020 ranges from 19% to 21%. The differences in the numbers of Chinese air freight in the second half of 2020 possible lies in the restrained belly cargo capacity for international passenger flights, as international air travel restrictions are assumed to be valid throughout the year.

Compared to China, air cargo transportation would have to endure a more considerable impact from their respective control measures in the long-term intervention scenario in the U.S. and Singapore in 2020 (Figs 5 and 6). For the U.S. air cargo industry, the start of the year has always been the lowest point annually, possibly explaining the drop in Jan and Feb of 2020, but the continuous plunges in Mar and Apr have to be explained by the impacts from the control measures. It must have been mainly because of the stay-at-home orders officially issued locally starting from mid Mar and elevated in Apr that drastically cut down the freight demand of businesses [50], in addition to which restrictions on traveling from the U.S. to other countries also played an role. Similar patterns apply to Singapore: a slight decrease in Jan and Feb, followed by a drastic plunge starting from Mar. Similar to the U.S., flight cancellations and the circuit breaker (CB) were issued in Mar and Apr in Singapore, respectively, and pertaining the same measures would keep its air freight at a low level (around 0.03 million tons monthly) till the yearend, incurring an annual loss of 0.664 million tons in air freight transportation (a percentage loss of 53.9%).

### Different impacts from short-term interventions

If the control measures could effectively contain the situation of COVID-19 within 6 months, releasing these measures and reactivating certain markets would be expected. If the control measures are enforced for 6 months and then released, air passenger numbers in the three selected countries would all be bounding up, but the shapes of the ‘V”s are different (see Fig 1). Provided that the control measures involving China were implemented strictly and promptly, the lowest air passenger number soon appeared in Feb, with an 85% decrease compared with 2019 Feb. Once the travel restrictions were fully released after 6 months (i.e. in Jul), people are expected to retaliatorily consume travel sources which would lead the number of air passengers to be 16% higher than normal level. The over-consumption would then disappear with a rationale market at the end the year and the air passenger level of China would return to a normal level with reasonable percentage deviations compared to a year earlier (see Table 7). A similar rebound is expected to occur to the air passenger numbers in the U.S., but the travel restrictions in the U.S. were less strict than those in China. Hence, there would be a less strong rebound in Sep and Oct. The predicted year-on-year percentage changes in U.S. air passengers also show that the numbers would rebound to a comparable level with the previous year from Oct with a deviation within 6% (see Table 7). Besides, as there is a relatively larger proportion of international travelers in the U.S. than in China, retaliating consumption is less likely to happen in the U.S. since international travel demands are relatively more rigid (i.e. for business or family return purposes). For Singapore, a rebound is also predicted to occur in Aug, but the number will not rebound to the normal level within this year, as the air passenger number in Singapore is highly related to the global situation of the pandemic.

Compared with the 6-month short-term intervention, the rebounding features of air passenger numbers of a 3-month intervention are different, mainly in China (see Fig 1). The retaliation in Chinese air passengers is weaker for a 3-month short-term confinement as the short period of restrictions in traveling has not yet resulted in strong demands of retaliatory consumptions. The year-on-year numbers in (see Table 6) indicates once the 3-month intervention ends, air passenger numbers in China would gradually increase but not able to reach the same level with last year till the end of 2020, leading to a totally 40% year-on-year decrease in 2020. For the U.S. and Singapore, the ‘V’ shapes of air passenger numbers are similar for 3-month and 6-month interventions. In the U.S., a strong rebound will occur in Jun after the restrictions are called off, reaching a higher number than normal; In Singapore, the number of air passenger would increase since May, but still unable to meet the normal level predicted by the SARIMA model (without interventions).

The rebound features of air freight of the three countries after a 6-month intervention reflect different impacts from control strictness at different levels. China, with comprehensively the strictest control measures (to deal with the most unknown epidemiological situation so far), has undergone a rather temporary impact on the air cargo level, and is expected to retrieve its normal air cargo level within two months once the control policies are canceled (Fig 4), rendering a relative decrease of 10.6% compared to the forecasts from the SARIMA results and a similarly 12% decrease compared with 2019. Comparatively, air transportation of goods in the U.S. and Singapore would be influenced for relatively longer time, although the number would finally rebound if the control policies are called off. Compared to the predicted air cargo intensities from the SARIMA models, the U.S. would have a 43% decline and Singapore would suffer a 36% decline under a 6-month intervention. In the case of Singapore, the tonnage of air freight is even more reluctant to rebound after a temporary (i.e. 3-month) intervention. In this sense, the strict and prompt responses from China has led to fewer losses in the air freight industry if the control policies are effective for 6 months.

## Discussions

National governments all over the world have been seeking ways to balance the losses in certain industries with the controlling effectiveness of the epidemic outbreak in the long run [1]. The three selected countries have implemented different strategies to control the spread of COVID-19. As a result, many industries are affected, including air transportation. The different patterns found in the results indicate that stricter and more effective measurements would possibly render larger impacts on the air transportation industry shortly, but the impact would be rather temporary and the long-term impact would be relatively milder (according to the predicted values of the two scenarios). Nonetheless, the short-term (and the long-term) intervention is a hypothesized scenario based on the assumption that the control measures are effective enough to suppress the epidemic to a certain level within half a year. If this is not the case, then either the constrain period would have to be prolonged, or stricter measures would have to be implemented [51], both of which are possible to cause more losses to the air transport industry in the long run.

Choices of the epidemic control policies and their impacts on the industry, and very possibly on the total economy, are found to be dependent with the certain features of the local market, and hence the best way to find a balance varies from country to country, from market to market. The case for China is the simplest: when the unfamiliar epidemic first raided its Wuhan City and disseminated fast to the whole country, it has quickly implemented relatively more strict control measures, having caused temporary drastic plunges in its air transportation industry. Since the measures were effective in controlling the epidemic, China gradually loosened the control measures and the passenger and cargo transport started to recover within two months. The next step for China is surely to gradually restore human mobility and freight transportation, under the condition that the importation and domestic transmission of COVID-19 is well controlled. The U.S. has a strong demand for related markets in both air passenger and freight transportation, and, at the same time, has a strong capability to serve the demand [46]. Hence, the most urgent task for the U.S. is to implement more strict measures to confine the virus from spreading domestically, possibly sacrificing the more industrial and economical revenues for a short period of time. Well-controlling the domestic epidemiological situation would enable the country to restore its consumer good demand and travel demand, so that the industry would rebound beyond normal levels.

Singapore has a very unique role in air transport in the world as a hub, and thus its Changi airport has an intensive schedule for serving inbound and outbound international flights [52, 53]. The impact from the control measures on air passengers and cargo transport in Singapore mainly lies in (1) the sharp cut in the capacity of international airlines and (2) the shrunken demand for consumer goods caused by the domestic CB. In this case, domestic epidemic control is only able to revive the demand, but the global pandemic situation would place a larger and wider impact on Singaporean air transportation. To balance the industrial losses and the development of COVID-19 situation, the Singaporean government is suggested to keep the pace to relax the CB and to introduce policies that may stimulate domestic consuming demands. On the supply side, airlines from low-risk countries could be conditionally open, yet the screening of the COVID-19 suspects should always be as strict as possible.

## Supporting information

S1 File Major control measures of China, Singapore and the U.S. with potential impacts on the air transportation industry. The three selected countries have implemented different types of control policies at distinct points of time. China has taken the lead to enforce cordon sanitaire and travel restrictions, followed by Singapore’s cutting inbound airline services responding to the global pandemic situation in Feb. The U.S. has actively issued flight cancellation policies at an early stage and less actively controlled domestic mobilities of people than the other two countries starting from Mar.
(PDF)

S2 File Decomposition results and Dickey-Fuller values of time series. (PDF)

S3 File The residual autocorrelation plots for the optimal intervention models. (PDF)

S1 Table *P* values for seasonal unit root (Dickey-Fuller) tests for all three countries. (PDF)

S2 Table Results of White Test. (PDF)

S3 Table Modeling results for SARIMA models for all three countries. (PDF)

S4 Table Comparison of the forecasting performance of 3-month short-term intervention models for all countries. Calculations of the performance metrics are based on the intervention period. The three-parameter form has a better performance in predicting intervention effects for air passengers in the U.S. and Singapore and air freight in the U.S.
(PDF)

S5 Table Comparison of the forecasting performance of 6-month short-term intervention models for all countries. Calculations of the performance metrics are based on the intervention period. The three-parameter form has a better performance in predicting intervention effects for air passengers in China and the U.S. and air freight in the U.S. and Singapore.
(PDF)

## References

[1] Guan DB and Wang DP and Hallegatte S and Davis SJ and Huo JW. Global supply-chain effects of COVID-19 control measures. Nature Human Behaviour. Nature Human Behaviour. 2020;4(6):577–87. 10.1038/s41562-020-0896-8 32493967

[2] Remuzzi A and Remuzzi G. COVID-19 and Italy: what next? The Lancet. 2020;395(10231):1225–1228. 10.1016/S0140-6736(20)30627-9 32178769 PMC7102589

[3] Wells CR and Sah P and Moghadas SM and Pandey A and Shoukat A. Impact of international travel and border control measures on the global spread of the novel 2019 coronavirus outbreak. Proceedings of the National Academy of Sciences of the United States of America. 2020;117(13):7504–7509. 10.1073/pnas.2002616117 32170017 PMC7132249

[4] WHO. Coronavirus disease 2019 (COVID-19) Situation Report. World Health Organization. 2020 Apr; Report No-74.

[5] Songhe Wang and Kangda Wei and Lei Lin and Weizi Li. Spatial-temporal analysis of COVID-19’s impact on human mobility: the case of the United States. 2020. arXiv, 2010.03707.

[6] Kang Y. and Gao S. and Liang Y. Multiscale dynamic human mobility flow dataset in the U.S. during the COVID-19 epidemic. Sci Data 7, 390 (2020). 10.1038/s41597-020-00734-5 33184280 PMC7661515

[7] Minha Lee and Jun Zhao and Qianqian Sun and Lei Zhang. Human mobility trends during the COVID-19 pandemic in the United States. 2020. arXiv, 2005.01215. 10.1371/journal.pone.0241468 PMC7652287 33166301

[8] Alessandro Galeazzi and Matteo Cinelli and Giovanni Bonaccorsi and Francesco Pierri. Human mobility in response to COVID-19 in France, Italy and UK. 2020. arXiv, 2005.06341. 10.1038/s41598-021-92399-2 PMC8222274 34162933

[9] Kraemer MUG and Yang CH and Gutierrez B and Wu CH and Klein B and Pigott DM. The effect of human mobility and control measures on the COVID-19 epidemic in China. Science. 2020;368(6490):493-+. 10.1126/science.abb4218 32213647 PMC7146642

[10] He GJ and Pan YH and Tanaka T. The short-term impacts of COVID-19 lockdown on urban air pollution in China. Nature Sustainability.

[11] IATA. Impact of COVID-19 on European Aviation. Geneva: 2020 June 20;Report No.

[12] International Air Transport Association [Internet]. Singapore. 2020; March 5.

[13] Shim E and Tariq A and Choi W and Lee Y and Chowell G. Transmission potential and severity of COVID-19 in South Korea. International Journal of Infectious Diseases. 2020;93:339–344. 10.1016/j.ijid.2020.03.031 32198088 PMC7118661

[14] Xu Shuojiang and Chan Hing Kai and Zhang Tiantian Forecasting the demand of the aviation industry using hybrid time series SARIMA-SVR approach. Transportation Research Part E: Logistics and Transportation Review. 2019 2;122: 169–180. 10.1016/j.tre.2018.12.005

[15] Milton Friedman. The interpolation of time series by related series. Journal of the American Statistical Association 57.300 (1962): 729–757. 10.1080/01621459.1962.10500812

[16] Gershenfeld N. The nature of mathematical modeling. New York: Cambridge University Press. pp. 205–208.

[17] Wang Yuanyuan and Wang Jianzhou and Zhao Ge and Dong Yao. Application of residual modification approach in seasonal ARIMA for electricity demand forecasting: A case study of China. Energy Policy. 2012 5; 48 284–294. 10.1016/j.enpol.2012.05.026

[18] Tsui Wai Hong Kan and Balli Hatice Ozer and Gilbey Andrew and Gow Hamish. Forecasting of Hong Kong airport’s passenger throughput. Tourism Management. 2014 4;42: 62–76. 10.1016/j.tourman.2013.10.008

[19] Holger Kantz and Schreiber Thomas. Nonlinear time series analysis. London: Cambridge University Press. pp. 185–201.

[20] Engle Robert F. Autoregressive conditional heteroscedasticity with estimates of the variance of United Kingdom inflation. Econometrica. 1982; 50(4): 987–1007. 10.2307/1912773

[21] Lu Zudi. A note on geometric ergodicity of autoregressive conditional heteroscedasticity (ARCH) model. Statistics and Probability Letters. 1996 3; 30(4): 305–311. 10.1016/S0167-7152(95)00233-2

[22] Mehdizadeh S. and Behmanesh J. and Khalili K. New approaches for estimation of monthly rainfall based on GEP-ARCH and ANN-ARCH hybrid models. Water Resource Manage. 2017 9; 32: 527–545. 10.1007/s11269-017-1825-0

[23] Neumeyer, N. and Omelka, M. and Hudecová, Š. A copula approach for dependence modeling in multivariate nonparametric time series. Journal of Multivariate Analysis. arXiv: 2018 Dec., 2019.

[24] Fearnhead Paul. Using random Quasi-Monte-Carlo within particle Filters, with application to financial time series. Journal of Computational and Graphical Statistics. 2005 1; 14(4): 751–769. 10.1198/106186005X77243

[25] Hörmann S. and Horváth L. and Reeder R. A functional version of the ARCH model. Econometric Theory. 2013 4; 29(2): 267–288. 10.1017/S0266466612000345

[26] Kim Jong-Min and Hwang Sun Young. Functional ARCH directional dependence via copula for intraday volatility from high-frequency financial time series. Applied Economics. 2020 9.

[27] Chris Brooks. Introductory econometrics for finance (3rd ed.). Cambridge: Cambridge University Press. pp. 461.

[28] Marek Lubos. 32nd International Conference on Mathematical Methods in Economics (MME). Olomouc, CZECH REPUBLIC. 2014 Sep; 10-12, 608-613.

[29] Min Jennifer C. H. and Lim C. and Kung H. Intervention analysis of SARS on Japanese tourism demand for Taiwan. Quality Quantity. 2011 10;45: 91–102. 10.1007/s11135-010-9338-4

[30] Joo Heesoo and Maskery Brian A. and Berro Andre D. and Rotz Lisa D. and Lee Yeon Kyeng. Economic Impact of the 2015 MERS Outbreak on the Republic of Korea’s Tourism-Related Industries. Health Security. 2019 4;17(2): 100–108. 10.1089/hs.2018.0115 30969152 PMC6560634

[31] Goh Carey and Law Rob. Modeling and forecasting tourism demand for arrivals with stochastic nonstationary seasonality and intervention. Tourism Management. 2002 10;23(5): 499–510. 10.1016/S0261-5177(02)00009-2

[32] Chen Ching-Fu and Chang Yu-Hern and Chang Yu-Wei. Seasonal ARIMA forecasting of inbound air travel arrivals to Taiwan. Transportmetrica. 2009 4;5(2), 125–140. 10.1080/18128600802591210

[33] Chang Yu Wei and Liao Meng Yuan. A seasonal ARIMA model of tourism forecasting: the case of Taiwan. Asia Pacific Journal of Tourism Research. 2010 3;15(2): 215–221. 10.1080/10941661003630001

[34] Zhang Xingyu and Zhang Tao and Young Alistair A. and Li Xiaosong. Applications and comparisons of four time series models in epidemiological surveillance data. PLOS ONE. 2014 2;2(9): 1–16. 10.1371/journal.pone.0088075 24505382 PMC3914930

[35] Gelhausen Marc C. and Berster Peter and Wilken Dieter. A new direct demand model of long-term forecasting air passengers and air transport movements at German airports. Journal of Air Transport Management. 2018 4;71, 140–152. 10.1016/j.jairtraman.2018.04.001

[36] Koc Erdogan and Altinay Galip. An analysis of seasonality in monthly per person tourist spending in Turkish inbound tourism from a market segmentation perspective. Tourism Management. 2007 2;28(1), 227–237. 10.1016/j.tourman.2006.01.003

[37] Phillips Peter C. B. and Xu Ke‐Li Inference in autoregression under heteroskedasticity. Journal of Time Series Analysis. 2006 3; 27(2): 289–308. 10.1111/j.1467-9892.2005.00466.x

[38] Box George E. P., Jenkins Gwilym M. Time series analysis: Forecasting and control. San Francisco: Holden Bay. 1976.

[39] Box G. E. P. and Tiao G. C. Intervention analysis with applications to economic and environmental problems. Journal of the American Statistical Association. 1975 10;70(349): 70–79. 10.1080/01621459.1975.10480264

[40] Li Shuying and Zhuang Jun and Shen Shifei. Dynamic forecasting conditional probability of bombing attacks based on time-series and intervention analysis. Risk Analysis. 2017 7;37(7): 1287–1297. 10.1111/risa.12679 27553923

[41] Chen Ying-Chih and Kang Hsin-Hong and Yang Tzer-Chyun. A study on the impact of SARS on the forecast of visitor arrivals to China. Journal of Asia-Pacific Business. 2008 10;8(1): 31–50. 10.1300/J098v08n01_04

[42] Payne James E. and Taylor J’Tia P. Modelling and forecasting airport passengers: a case study for an introductory forecasting course. International Journal of Information and Operations Management Education. 2007 10;2(2): 167–182. 10.1504/IJIOME.2007.015282

[43] Tang B and Wang X and Li Q and Bragazzi NL and Tang SY and Xiao YN. Estimation of the transmission risk of the 2019-nCoV and its implication for public health interventions. Journal of Clinical Medicine. 2020;9(2). 10.3390/jcm9020462 32046137 PMC7074281

[44] Xu B and Gutierrez B and Mekaru S and Sewalk K and Goodwin L and Loskill A. Epidemiological data from the COVID-19 outbreak, real-time case information. Scientific data. 2020;7(1):1–6. 10.1038/s41597-020-0448-0 32210236 PMC7093412

[45] Institution QIR. Report of the development of civil airline industry in China, 2019. 2020 Feb 20. Report No.

[46] Airfarewatchdog. COVID-19 flight cancellations by region and airline: airfarewatchdog. 2020.

[47] Statistics BoT. Annual U.S. Domestic Average Itinerary Fare in Current and Constant Dollars. Washington D.C., USA: 2020.

[48] Purchasing CFoL. Statistics for logistics operation in China. Beijing: Ministry of Civil Affairs of China, 2020.

[49] Growth Insights on China’s Pharmaceutical Industry (2020 to 2025). DUBLIN: 2020.

[50] See Which States and Cities Have Told Residents to Stay at Home [Internet]. New York; 2020; Apri 20. Available from: https://www.nytimes.com/interactive/2020/us/coronavirus-stay-at-home-order.html

[51] Chinazzi M and Davis JT and Ajelli M and Gioannini C and Litvinova M and Merler S. The effect of travel restrictions on the spread of the 2019 novel coronavirus (COVID-19) outbreak. Science. 2020;368(6489):395-+. 10.1126/science.aba9757 32144116 PMC7164386

[52] Wu HJ and Tsui KWH. Does a reward program affect customers’ behavioural intention of visiting the airport? A case study of Singapore Changi Airport. Journal of Air Transport Management. 2020;82. 10.1016/j.jairtraman.2019.101742

[53] Chang YC and Lee WH, Hsu CJ. Identifying competitive position for ten Asian aviation hubs. Transport Policy. 2020;87:51–66. 10.1016/j.tranpol.2020.01.003

