# Supplementary material for: Impact of different control policies for COVID-19 outbreak on the air transportation industry: A comparison between China, the U.S. and Singapore
Source: PLoS One. 2021 Mar 16;16(3):e0248361. doi: 10.1371/journal.pone.0248361 (PMC7963044; doi:10.1371/journal.pone.0248361)
Supplement: S1 File — The three selected countries have implemented different types of control policies at distinct points of time. China has taken the lead to enforce cordon sanitaire and travel restrictions, followed by Singapore’s cutting inbound airline services responding to the global pandemic situation in Feb. The U.S. has actively issued flight cancellation policies at an early stage and less actively controlled domestic mobilities of people than the other two countries starting from Mar. (PDF) [file pone.0248361.s001.pdf]

S1 File. Major control measures of China, Singapore and the US with potential impact on the air transportation industry.

Month	China	Singapore	US
Jan	22nd: Hubei Province activated Level II emergent response to public health event. Chinese State Council announced to prolong Chinese New Year vacation. Civil Aviation Administration of China (CAAC) issued policies for ticket refund.<br>23rd: human-to-human transmission was confirmed, and Wuhan city was locked down. Airports and train stations were closed temporarily.<br>24th: The Ministry of Culture and Tourism instructed that tourism agencies should suspend providing tourism services. Touristy spots and cultural and entertainment places suspended their operations nationwide.<br>27th: Ministry of Education instructed universities, high schools and middle schools to postpone new semesters.	23rd – 31st: airlines including Scoot, Singapore Airlines, SilkAir etc. reduced or canceled flights to and from certain areas in China with high risks.	31st: President Trump issued an order restricting travel to the US from China.
Feb		18th: Singapore Airline and Silkair announced plans for massive international flight cancellations over the network.	
Mar	12th: CAAC controlled the total number of international arriving passenger flights.<br>26th: CAAC announced "Five Ones" policy.	3rd: travel advisories were issued by the Ministry of Foreign Affairs targeting at banning visitors from South Korea, Iran, Northern Italy, etc.<br>29th: approvals were required for long-term pass holders before entering Singapore.	11th: President Trump issued an order restricting travel to the US from certain European countries with high risk levels. Universities and colleges suspended in-person classes.<br>15th: The Center for Disease Control (CDC) issued guidance advocating against social gatherings. President Trump issued new guidelines urging people to avoid discretionary travel.<br>23rd: stay-at-home orders were issued in 20 states either statewide or in part of the state.
Apr		3rd: circuit breaker (CB) was initially enforced with control measures including closing non-essential workplaces, schools transitioned to home-based learning.<br>21st: an tightened CB was announced by the Prime Minister with restriction to entering certain retail franchises.	
May		1st: Terminal 2 of Changi Airport started to be suspended for 18 months.<br>2nd: the CB was relaxed by reopening tradition Chinese medicine shops and essential condo activities.<br>16th: Terminal 4 of Changi Airport started to be temporarily suspended.	
