# Supplementary material for: Impact of different control policies for COVID-19 outbreak on the air transportation industry: A comparison between China, the U.S. and Singapore
Source: PLoS One. 2021 Mar 16;16(3):e0248361. doi: 10.1371/journal.pone.0248361 (PMC7963044; doi:10.1371/journal.pone.0248361)

S2 File. Decomposition results and Dickey-Fuller values of time series.

China Air Passengers

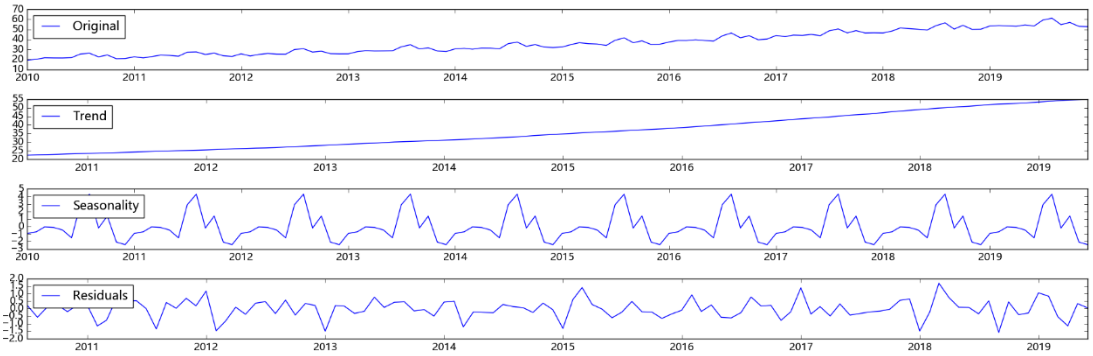

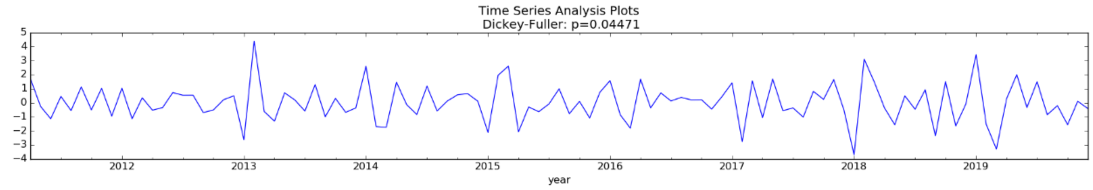

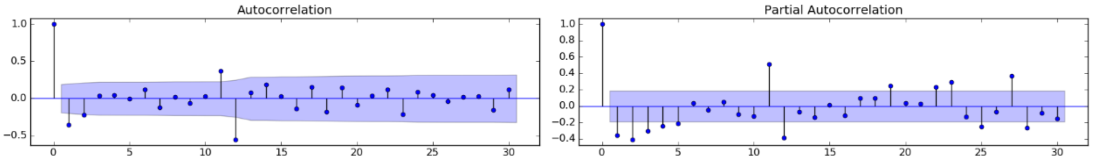

China Air Freight

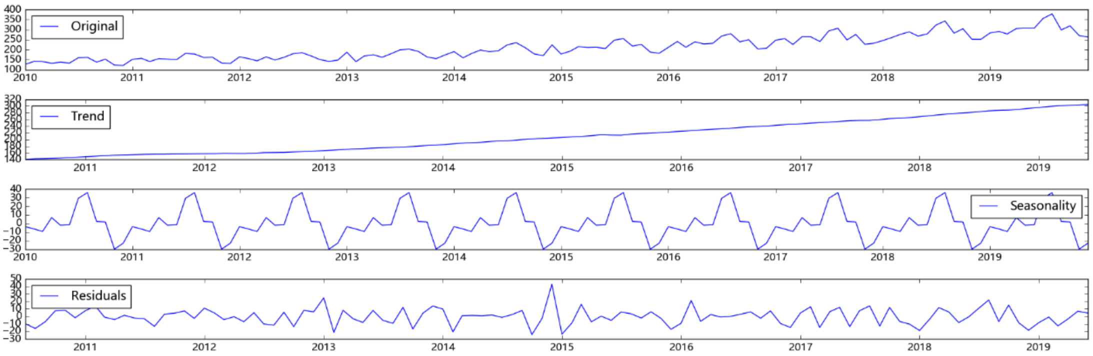

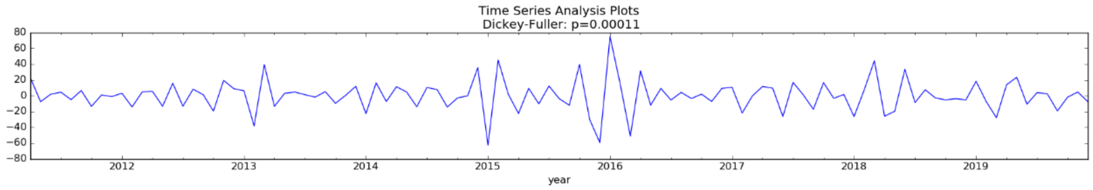

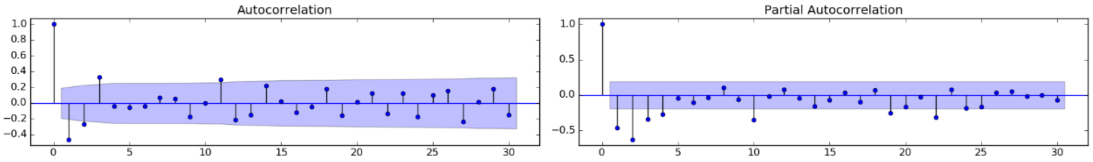

U.S. Air Passengers

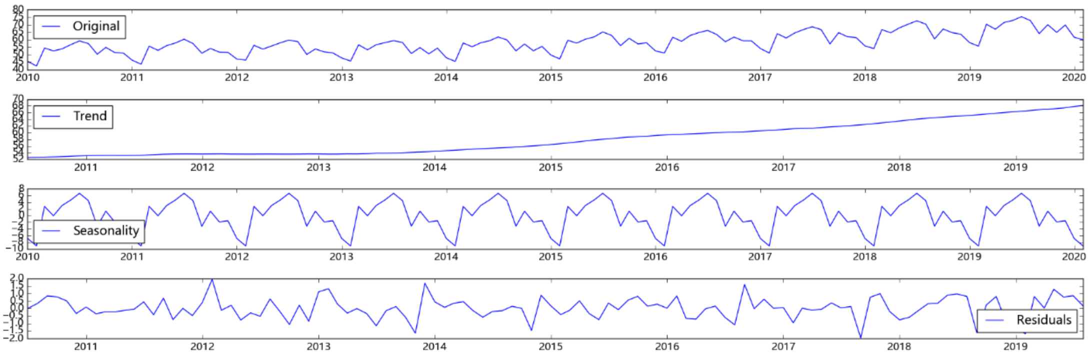

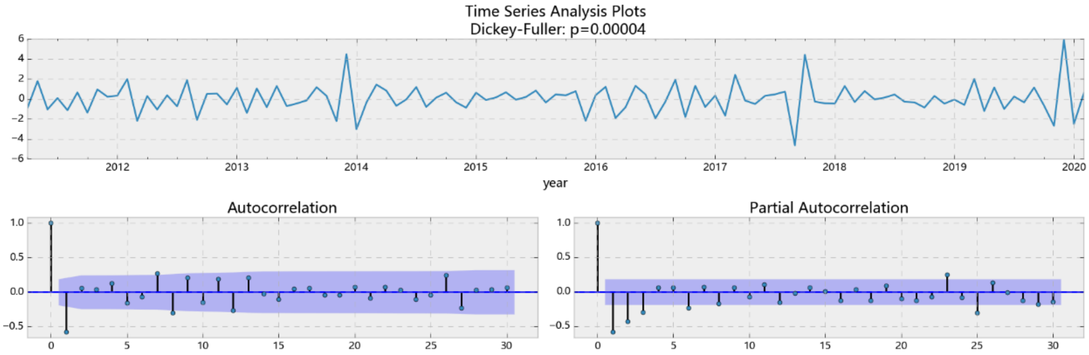

U.S. Air Freight

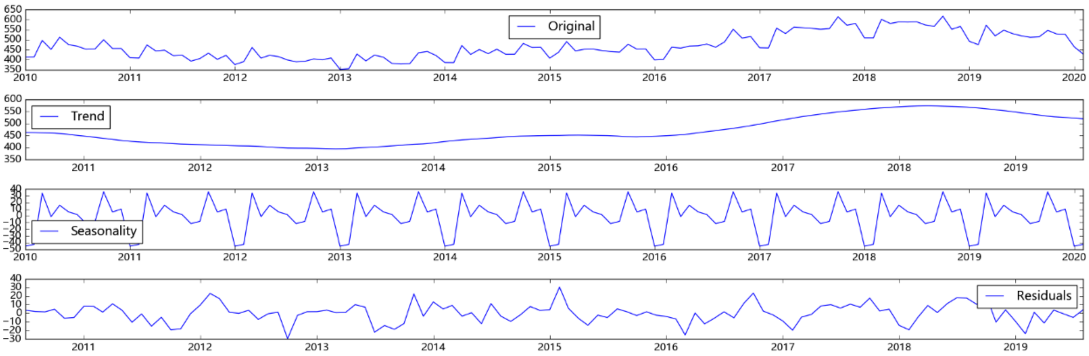

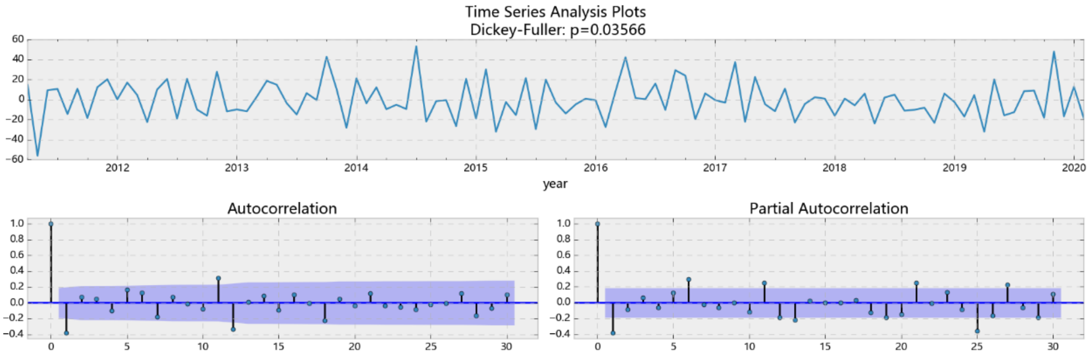

Singapore Air Passengers

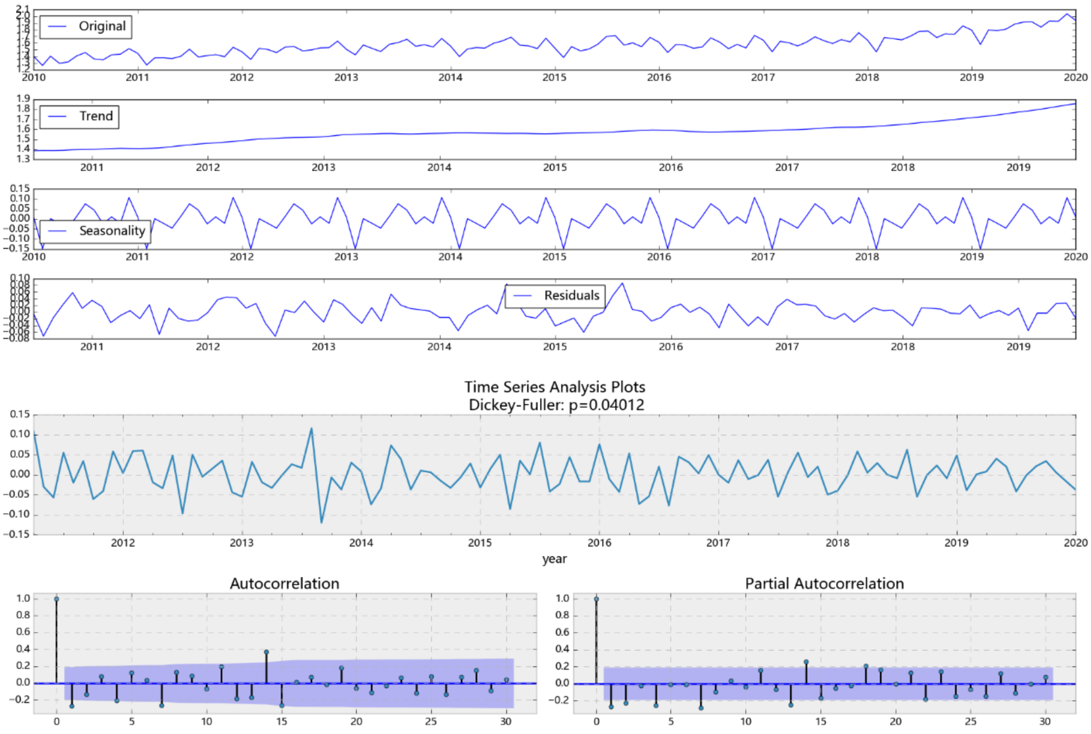

Singapore Air Freight

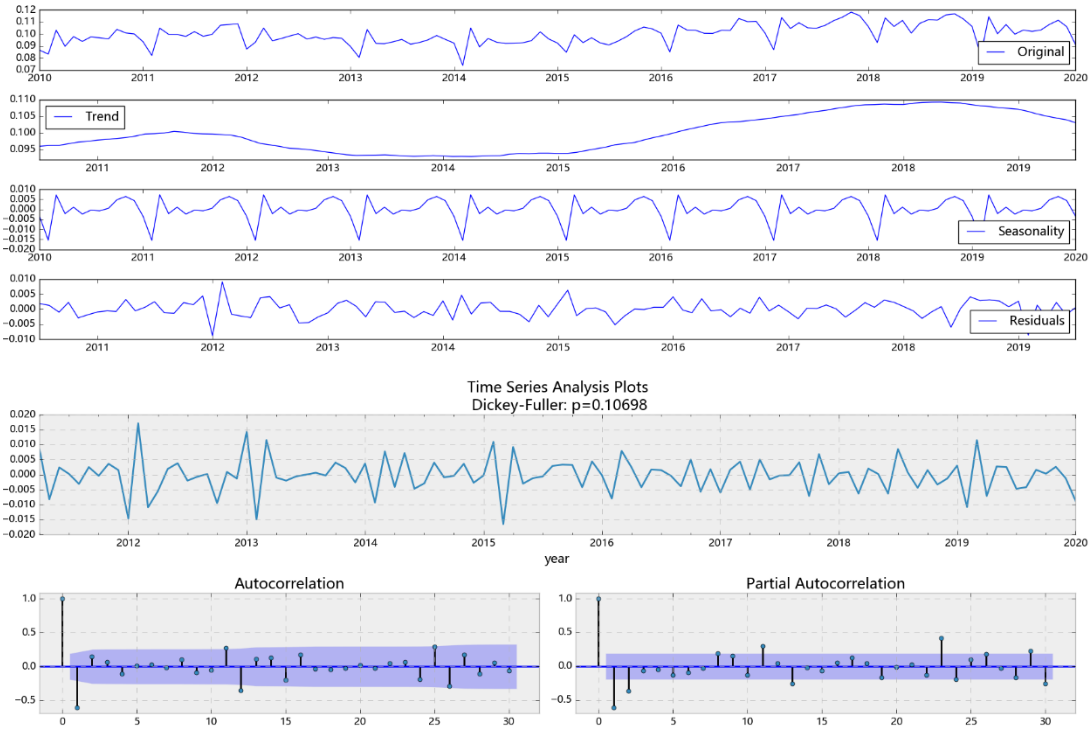

Supplement: S2 File — (PDF) [file pone.0248361.s002.pdf]
