# Supplementary material for: Impact of different control policies for COVID-19 outbreak on the air transportation industry: A comparison between China, the U.S. and Singapore
Source: PLoS One. 2021 Mar 16;16(3):e0248361. doi: 10.1371/journal.pone.0248361 (PMC7963044; doi:10.1371/journal.pone.0248361)
Supplement: S3 File — (PDF) [file pone.0248361.s003.pdf]

S3 File. The residual autocorrelation plots for the optimal intervention models.

Long-term intervention:

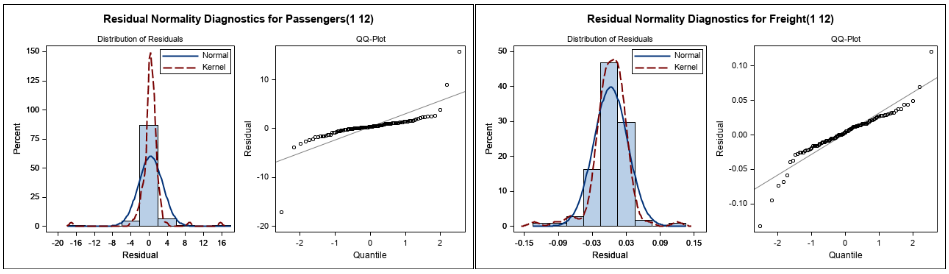

China

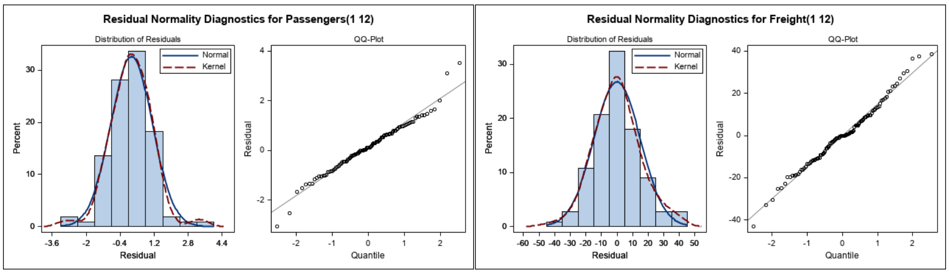

U.S.

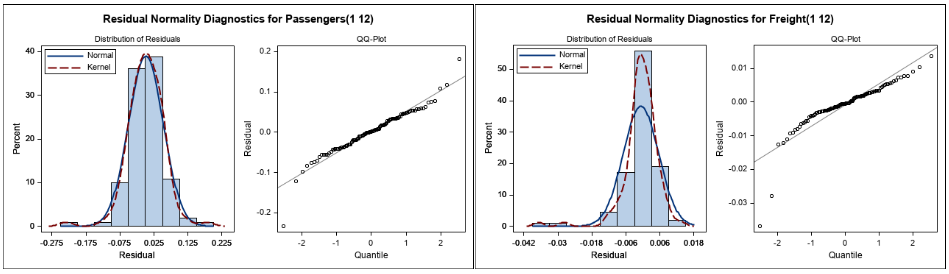

Singapore

6-month intervention

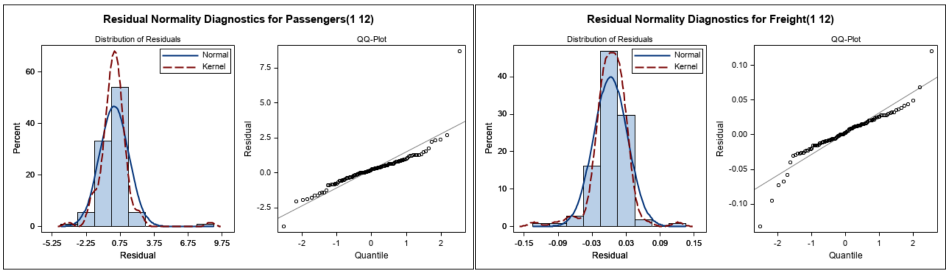

China

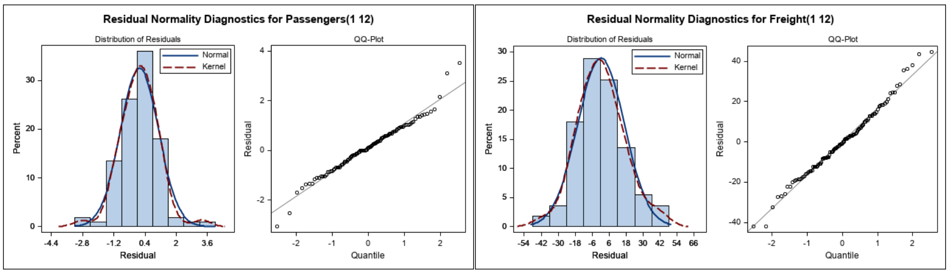

U.S.

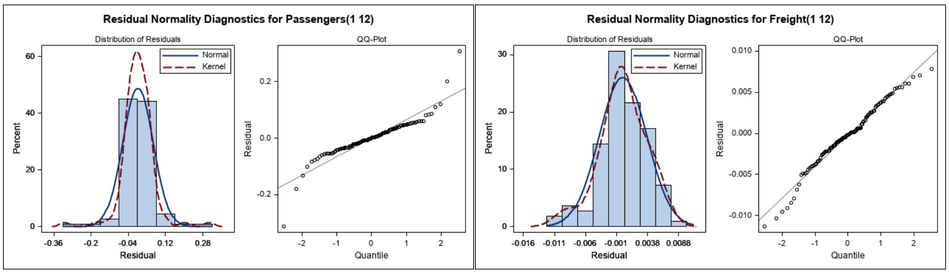

Singapore

3-month intervention

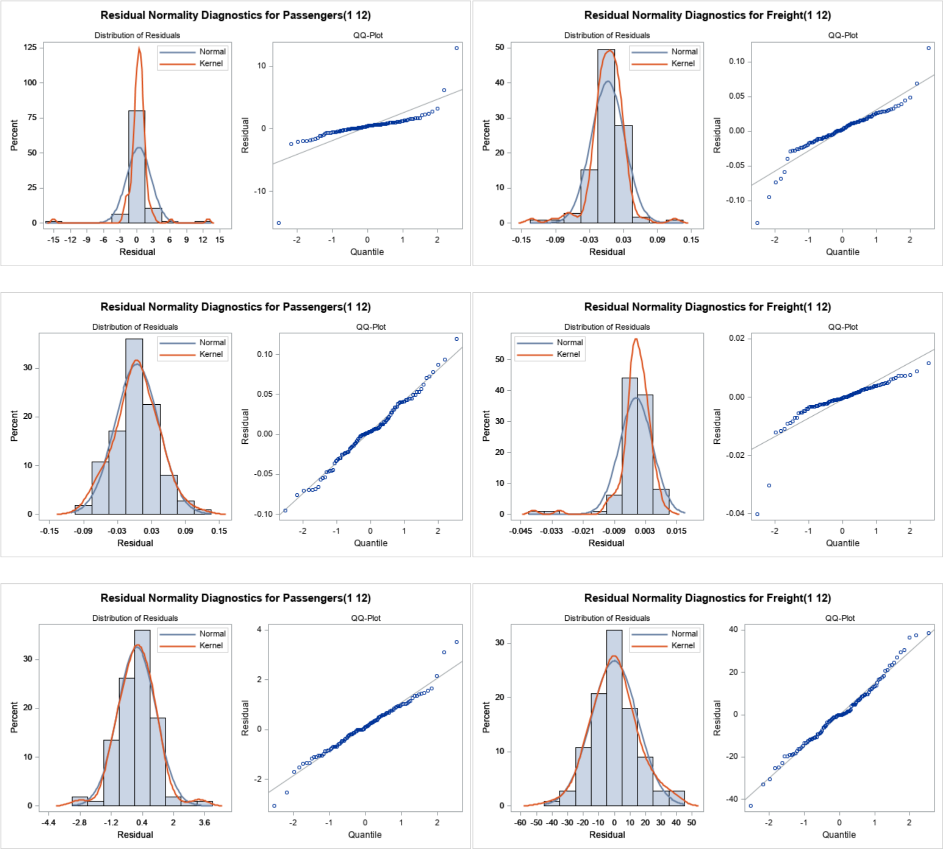

China

U.S.

Singapore
