# Supplementary material for: Impact of different control policies for COVID-19 outbreak on the air transportation industry: A comparison between China, the U.S. and Singapore
Source: PLoS One. 2021 Mar 16;16(3):e0248361. doi: 10.1371/journal.pone.0248361 (PMC7963044; doi:10.1371/journal.pone.0248361)
Supplement: S1 Table — (PDF) [file pone.0248361.s004.pdf]

S1 Table. P values for seasonal unit root (Dickey-Fuller) tests for all three countries.

**Indicators**	**China**		**U.S.**		**Singapore**	
	Air passengers	Air freight	Air passengers	Air freight	Air passengers	Air freight
Unit root test (Dickey-Fuller)	0.119	0.538	0.148	0.0618	0.639	0.593
