# Supplementary material for: Impact of different control policies for COVID-19 outbreak on the air transportation industry: A comparison between China, the U.S. and Singapore
Source: PLoS One. 2021 Mar 16;16(3):e0248361. doi: 10.1371/journal.pone.0248361 (PMC7963044; doi:10.1371/journal.pone.0248361)
Supplement: S2 Table — (PDF) [file pone.0248361.s005.pdf]

S2 Table. Results of White Test.

Indicators	China		U.S.		Singapore	
	Air passengers	Air freight	Air passengers	Air freight	Air passengers	Air freight
Lagrange Multiplier Statistic p value	0.985	0.209	0.986	0.969	0.735	0.976
F-Statistic p value	0.986	0.215	0.986	0.061	0.742	0.977
