# Supplementary material for: Impact of different control policies for COVID-19 outbreak on the air transportation industry: A comparison between China, the U.S. and Singapore
Source: PLoS One. 2021 Mar 16;16(3):e0248361. doi: 10.1371/journal.pone.0248361 (PMC7963044; doi:10.1371/journal.pone.0248361)
Supplement: S3 Table — (PDF) [file pone.0248361.s006.pdf]

S3 Table. Modeling results for SARIMA models for all three countries.

Indicators	Estimated coefficient			Model performance			
	AR(1)	MA(1)	SMA(12)	Adj-R^2	AIC	BIC	Q-statistics
China air passengers	-	-0.6964	-0.5274	0.879	167.9	174.4	0.55
U.S. air passengers	-	-0.6302	-1.1878	0.821	169.1	175.6	0.18
Singapore air passengers	0.3946	-0.8846	-	0.554	-255.6	-248.7	0.30
China air freight	-0.4210	-0.8134	-	0.671	-300.2	-293.3	0.18
U.S. air freight	-0.3092	-	-0.7498	0.649	545.8	552.3	0.31
Singapore air freight	-	-0.6434	-0.5009	0.589	-521.1	-514.7	0.93

D and d are both equal to 1, and all estimated coefficients are significant at the 0.05 level or above.
