# Supplementary material for: Impact of different control policies for COVID-19 outbreak on the air transportation industry: A comparison between China, the U.S. and Singapore
Source: PLoS One. 2021 Mar 16;16(3):e0248361. doi: 10.1371/journal.pone.0248361 (PMC7963044; doi:10.1371/journal.pone.0248361)
Supplement: S4 Table — Calculations of the performance metrics are based on the intervention period. The three-parameter form has a better performance in predicting intervention effects for air passengers in the U.S. and Singapore and air freight in the U.S. (PDF) [file pone.0248361.s007.pdf]

S4 Table. Comparison of the forecasting performance of 3-month short-term intervention models for all countries. Calculations of the performance metrics are based on the intervention period. The three-parameter form has a better performance in predicting intervention effects for air passengers in the U.S. and Singapore and air freight in the U.S.

**Indicators**	**Linear**			**Non-linear two-parameter**			**Non-linear three-parameter**		
	MAPE (%)	MAE	RMSE	MAPE (%)	MAE	RMSE	MAPE (%)	MAE	RMSE
China air passengers	43.18	25.54	27.071	18.12	3.68	4.82	-	-	-
U.S. air passengers	-	-	-	-	-	-	0.00061	0.00011	0.00015
Singapore air passengers	-	-	-	-	-	-	63.36	0.25	0.26
China air freight	13.94	0.091	0.098	6.16	0.028	0.038	-	-	-
U.S. air freight	31.68	152.44	157.028	15.76	37.092	38.13	2.39	11.12	14.74
Singapore air freight	22.22	0.021	0.027	8.81	0.0045	0.0052	-	-	-
