# Supplementary material for: Impact of different control policies for COVID-19 outbreak on the air transportation industry: A comparison between China, the U.S. and Singapore
Source: PLoS One. 2021 Mar 16;16(3):e0248361. doi: 10.1371/journal.pone.0248361 (PMC7963044; doi:10.1371/journal.pone.0248361)
Supplement: S5 Table — Calculations of the performance metrics are based on the intervention period. The three-parameter form has a better performance in predicting intervention effects for air passengers in China and the U.S. and air freight in the U.S. and Singapore. (PDF) [file pone.0248361.s008.pdf]

S5 Table. Comparison of the forecasting performance of 6-month short-term intervention models for all countries. Calculations of the performance metrics are based on the intervention period. The three-parameter form has a better performance in predicting intervention effects for air passengers in China and the U.S. and air freight in the U.S. and Singapore.

Indicators	Linear			Non-linear two-parameter			Non-linear three-parameter		
	MAPE (%)	MAE	RMSE	MAPE (%)	MAE	RMSE	MAPE (%)	MAE	RMSE
China air passengers	30.32	15.69	17.027	18.95	3.45	4.77	17.12	2.97	4.31
U.S. air passengers	-	-	-	0.015	0.017	0.0032	0.00061	0.00011	0.00015
Singapore air passengers	92.68	0.22	0.23	19.64	0.25	0.26	-	-	-
China air freight	13.062	0.079	0.082	5.096	0.023	0.027	-	-	-
U.S. air freight	48.17	105.31	111.85	15.76	37.092	38.13	2.39	11.12	14.74
Singapore air freight	24.64	0.021	0.026	12.33	0.0071	0.0073	3.17	0.0029	0.0038
